# Invisibility and interpretation

**DOI:** 10.3389/fpsyg.2014.00975

**Published:** 2014-09-17

**Authors:** Michael H. Herzog, Frouke Hermens, Haluk Öğmen

**Affiliations:** ^1^Brain Mind Institute, Ecole Polytechnique Fédérale de Lausanne (EPFL)Lausanne, Switzerland; ^2^School of Psychology, University of AberdeenAberdeen, UK; ^3^Department of Electrical and Computer Engineering, Center for NeuroEngineering and Cognitive Science, University of HoustonHouston, TX, USA

**Keywords:** masking, priming, visibility, consciousness

## Abstract

Invisibility is often thought to occur because of the low-level limitations of the visual system. For example, it is often assumed that backward masking renders a target invisible because the visual system is simply too slow to resolve the target and the mask separately. Here, we propose an alternative explanation in which invisibility is a goal rather than a limitation and occurs naturally when making sense out of the plethora of incoming information. For example, we present evidence that (in)visibility of an element can strongly depend on how it groups with other elements. Changing grouping changes visibility. In addition, we will show that features often just appear to be invisible but are in fact visible in a way the experimenter is not aware of.

## 1. Introduction

It is often assumed that invisibility reflects fundamental limitations of the human visual system and, vice versa, any stimulus that is above these limitations becomes automatically visible. For example, objects that are too small with respect to the spatial resolution of the visual system are invisible but can be clearly perceived after magnification by a microscope. However, there are interesting cases where a stimulus is visible when presented alone but becomes invisible when it is presented with other stimuli. Here, the stimulus itself is clearly above the basic resolution limits of the visual system. There are two cases. The first case is based on rather low-level “irreversible” automatic mechanisms. For example, a clearly visible but faint line becomes invisible in the neighborhood of a high luminance patch because of gain control. Similarly, the stars are invisible during the day because the bright sunlight drives the rod system into saturation. In these cases, invisibility occurs because of adjusting the dynamic range of the visual system to that of the environment. However, we claim that there are many situations, frequently used in consciousness research and many other fields, where invisibility is not caused by such low-level– and irreversible– factors, in the sense that “flexible” changes in context can lead to drastic changes in visibility. Here, we show evidence for this second type of invisibility by the example of visual masking but argue that similar processes of Gestalt formation play a central role for (in)visibility in general.

In masked priming studies, a target is followed by a mask (Klotz and Neumann, 1999; Schmidt, 2002; Vorberg et al., 2003). In a first condition, observers are asked to discriminate the features of the target (direct measure). If parameters of the target and mask are well chosen, performance on the target is at or close to chance level, i.e., its features are unconscious and invisible. Still, the features of the target can *prime* response times when observers discriminate, in a second condition, features of the mask (indirect measure). We like to mention that in most of these studies only the *features* of the target are unconscious, and not the target *itself*. The priming effects are often explained by pre-activation of the motor system (e.g., Klotz and Neumann, 1999; Schmidt, 2002; Vorberg et al., 2003). In a fast, unconscious processing sweep, the target pre-activates the motor cortex, which leads to speeded or slowed processing when the task is on the mask. Support for this motor pre-activation comes from many behavioral (Schmidt, 2002) and ERP studies (e.g., Eimer and Schlaghecken, 2003). However, why are the target features invisible at all? Invisibility is usually thought to occur because of the limitations of the visual system. For example in integration masking, it is assumed that target and mask integrate into *one* conglomerate because the visual system cannot separate the two stimuli due to its limited temporal resolution. Integration may well occur as early as on the retinal level and can be seen as a superposition of the target and mask, similar to when two slides are projected together (e.g., Di Lollo, 1980; Kahneman, 1968; Scheerer, 1973). Hence, invisibility occurs because the system is at its temporal limits. Other mechanisms rely on different types of masking and other types of low level mechanisms such as in metacontast masking (see Discussion).

Here, we show evidence that invisibility is often not the outcome of limitations of the visual system. Instead, we argue that invisibility is due to purposeful interpretation, i.e., a goal rather than a limitation. Visibility and invisibility depend on how elements group and occur naturally and necessarily because of the ill-posed problems of vision.

## 2. Review of evidence

### 2.1. Excellent temporal precision

**Figure 1:**
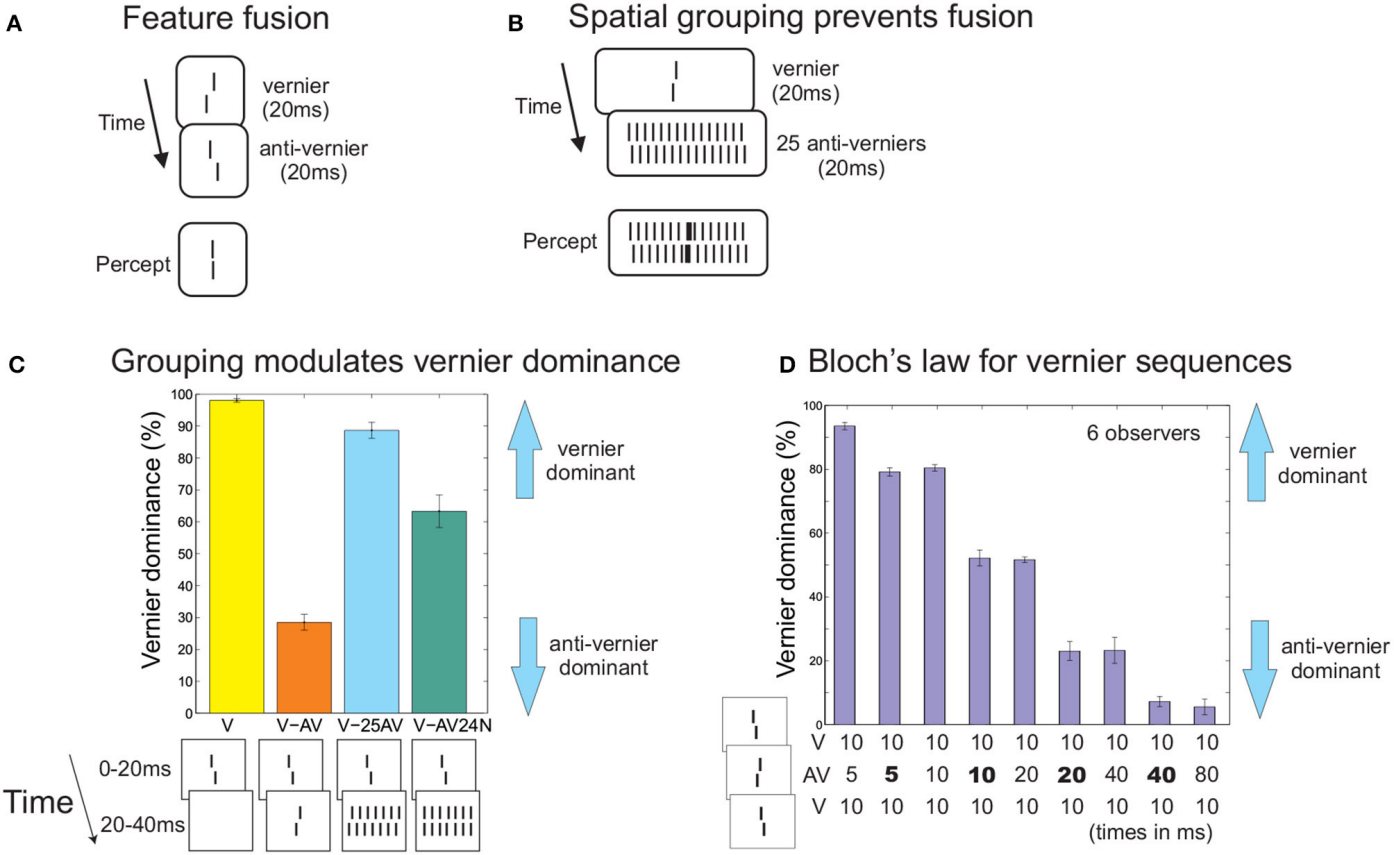
**(A)** Feature fusion. A right or left vernier (V) is followed by a second vernier with an opposite offset direction (anti-vernier, AV). Only one vernier with an almost aligned offset is perceived. Observers cannot tell whether the first or second vernier is offset to the right. Hence, the verniers themselves are unconscious (direct measure of awareness). **(B)** When the second vernier is flanked by 12 copies of itself on each side, temporal feature fusion almost completes ceases. The first vernier becomes visible as an element in its own right, appearing to be superimposed on the grating of 25 anti-verniers. **(C)** To give quantitative expression, we determined vernier dominance. In each trial, we determined whether the response of the observers matched the offset direction of the first vernier. Hence, a 100% score means that observers pushed always the button according to the first vernier. A 0% score means observers decided always according to the second vernier, and for 50% both verniers contributed equally on average. Performance is at ceiling for a single vernier (V). Adding the anti-vernier leads to anti-vernier dominance (in accordance with the fact that in fusion, the latter element dominates). When there are 2^*^12 copies of the second vernier, the first vernier becomes visible and performance is almost as good as for the single vernier (V-25AV; only 2^*^6 verniers are shown here). Locally, at the center, the very same first and second vernier are presented as in the previous conditions. Performance is roughly independent of whether or not these verniers are offset or aligned indicating again that there is no temporal fusion (V-AV24N). We argue that spatial integration trumps temporal fusion. Visibility of the first vernier depends on grouping and not on low level limitations. Data from Hermens et al. (2009). **(D)** Bloch's law for 3-vernier sequences. Doubling the luminance of the anti-vernier has the same effect as doubling its duration (doubled luminances are indicated by bold font). Data from Scharnowski et al. (2007a).

Consider the situation in Figure 1A. A right offset vernier is followed immediately by a left offset vernier or vice versa. An almost aligned vernier is perceived, a phenomenon called feature fusion (Efron, 1967; Herzog et al., 2003). Participants cannot perceive the individual verniers^1^. This outcome is classically explained by integration masking where the two verniers are invisible because of a lack of temporal resolution (Scheerer, 1973; Turvey, 1973). The human brain is simply not *able* to render the two elements visible individually.

However, this is not the case. When we presented an array of 25 verniers in the second frame, the first vernier became visible and discrimination of its offset was only slightly deteriorated compared to when presented alone (Figures 1B,C; Hermens et al., 2009). Hence, even though the two central verniers are identical to the feature fusion condition, the human brain can now easily resolve the two verniers and their offsets. We propose that the 25 verniers are grouped as *one* array of elements independently of the first vernier, which is thus rendered visible as an element in its own right (Herzog and Koch, 2001; Herzog and Fahle, 2002; Ghose et al., 2013). In line with this proposition, the offset direction of the 25 verniers has almost no influence, i.e., performance is roughly the same when the 25 verniers are offset in the opposite direction than the first vernier or not offset at all (Hermens et al., 2009). Spatial grouping of the vernier array prevents the *temporal* integration of the first and second vernier, even though both are presented at the same retinal location in both conditions. This suggests that (in)visibility depends crucially on perceptual grouping.

Next, we show that the integration of vernier offsets is extremely precise, meaning that while participants cannot report the individual verniers, information about their individual offsets, durations and luminance is still preserved. We presented three verniers in immediate succession. The first and last vernier had always the same offset direction (left or right), whereas the second vernier was offset in the opposite direction. Presentation time was 10 ms for the first and last vernier, and varied for the second vernier. When the second vernier's duration was 5 ms, performance was strongly determined by the first (and third) vernier (Figure 1D). When we doubled the luminance of the second vernier, still presented for 5 ms, it contributed more strongly to performance (Figure 1D), meaning that although participants could not tell how many verniers were presented and of what luminance, the brain still incorporates this information. Performance remained on this level when we increased the duration to 10 ms but reduced the luminance to normal. Hence, increasing/decreasing the duration can be compensated by decreasing/increasing the luminance. Feature fusion follows precisely Bloch's law, which states that brightness is the product of luminance times duration (Scharnowski et al., 2007a). Hence, we have shown that even though the individual vernier offsets are invisible, the human brain carefully analyzes and integrates them into one offset, which in turn is consciously perceived. This means that in feature fusion, the outputs of specific feature detectors (vernier offsets) are combined into a meaningful percept, depending on perceptual grouping. This is very different from integration masking, in which integration results in a “pixel-by-pixel” conglomerate of the target and the mask (on the retinal level), similar to when two slides are projected together superimposed.

Here is another illustration of why feature fusion differs fundamentally from integration masking. In the case of feature fusion with the two verniers (Figure 1C), performance is close to the 50% level with a slight dominance of the second vernier. This result may be taken as chance level performance because of integration masking (the superposition of a first right and second left vernier is the same as the other way around and, hence, observers cannot discriminate the two situations). However, as we have argued, the 50% level just shows that the two vernier offsets are equally weighted in the integration. Performance quickly changes from 50% when one of the verniers has a slightly higher luminance (or offset size). Similar results were found for complex sequences of verniers (Hermens et al., 2010).

Feature fusion occurs also in other visual domains. For example, when a red disk is followed by a green disk, only one yellow disk is perceived (Efron, 1967, 1973). Also here, while the individual elements (the disks) are not visible to the participants, the features of the elements are still registered by the brain and combined into *one* perceived object.

### 2.2. Unconsciousness and long lasting feature integration

Up to now, we have provided evidence that invisibility can be the result of grouping rather than of low level limitations of the visual system. Here, we show, first, that the vernier offsets are indeed unconscious. Second, the unconscious offsets are represented in the visual system for an extensive duration (of around 420 ms) even though the individual stimuli (vernier and anti-vernier) are presented for a short time (30 ms each).

We presented a vernier and the anti-vernier, as above. Observers were told about the set-up and asked whether the first or second vernier is offset to the right (Scharnowski et al., 2009). Performance was at about 52%, i.e., almost at chance level. As an aside note, we use two direct measures to test for unconsciousness (“which vernier is offset to the right” and “what is the conscious, fused vernier offset”) rather than a direct and an indirect one (based on reaction times, as in priming studies, e.g., Schmidt, 2002; Vorberg et al., 2003).

**Figure 2:**
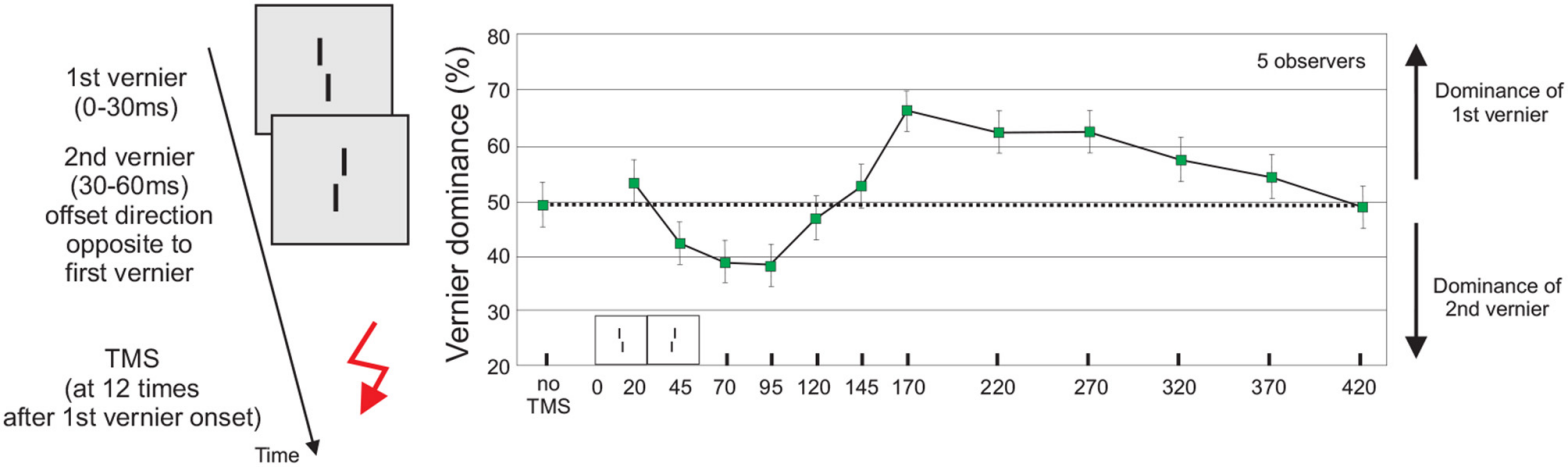
**The influence of TMS on feature fusion**. A sequence of a vernier and an anti-vernier is followed by a TMS-pulse to the occipital cortex. The offsets of the vernier and anti-vernier are adjusted so that for each participant, vernier dominance is at 50% without TMS (dotted line). TMS applied at SOAs 20–120 ms leads to dominance of the second vernier, TMS applied at SOAs 120–420 ms leads to dominance of the first vernier. The long-lasting TMS effect indicates that feature integration cannot be completed before 420 ms. Figure adapted from Scharnowski et al. (2009). The arrow indicates the TMS pulse.

Next, we show that the unconscious vernier offsets are not only carefully analyzed and integrated but that this process lasts for up to 420 ms after stimulus onset. We presented the first and second vernier for 30 ms each. We adjusted the second vernier offset such that dominance was at about 50% for each participant. Hence, both verniers contributed equally to feature fusion (Figure 2). Next, we applied Transcranial Magnetic Stimulation (TMS) at various stimulus onset asynchronies (SOAs) over the occipital lobe. For SOAs up to 120 ms, TMS led to dominance of the second vernier. From 120 to 420 ms, the first vernier dominated. The most important conclusion from this finding is that the two verniers cannot be fully integrated before 420 ms for the following reason. By design, both verniers contribute equally to fusion in the basic no-TMS-condition. When the verniers were integrated immediately, TMS could only modulate the combined representation but not favor one vernier over the other. The trick of the experiment is that the 50% dominance level (equal contribution of both verniers to fusion) and chance level performance are the same. In this sense, whatever the effect of TMS on the integrated representation is, discrimination of its offset remains at chance (similar to the outcome when observers would close their eyes). Now by the converse argument, if performance is *not* at 50%, TMS “favors” one vernier over the other and hence the two cannot be fully integrated, i.e., there are separate representations for the two verniers for at least 420 ms (Scharnowski et al., 2009). As an implication, this experiment provides evidence that conscious access to feature information in our paradigm cannot emerge before 420 ms, since the vernier offsets themselves are unconsciousness as shown above.

We like to add that vernier fusion, as well as color and motion fusion, can be manipulated also by light masks, instead of TMS (Pilz et al., 2013).

### 2.3. Feature integration across space and time

Up to now, we have shown that, first, whether an element is rendered visible or invisible can crucially depend on perceptual grouping. In these instances, invisibility cannot be explained by limited temporal resolution or other low-level mechanisms. Second, we have provided evidence that features even of invisible elements are carefully registered and, depending on grouping, integrated with other elements. Third, we have shown that unconscious feature integration can be a long lasting process. Using TMS, we found that consciousness does not emerge before 420 ms.

The question arises, why is feature integration so long lasting? Why is there is such a long period of unconsciousness? Here, we will suggest that the brain needs to integrate information across extended periods because we and the world are in permanent motion. In addition, we will show that features of an invisible element can be rendered visible at other elements. Hence, features of elements can be transported across space.

**Figure 3:**
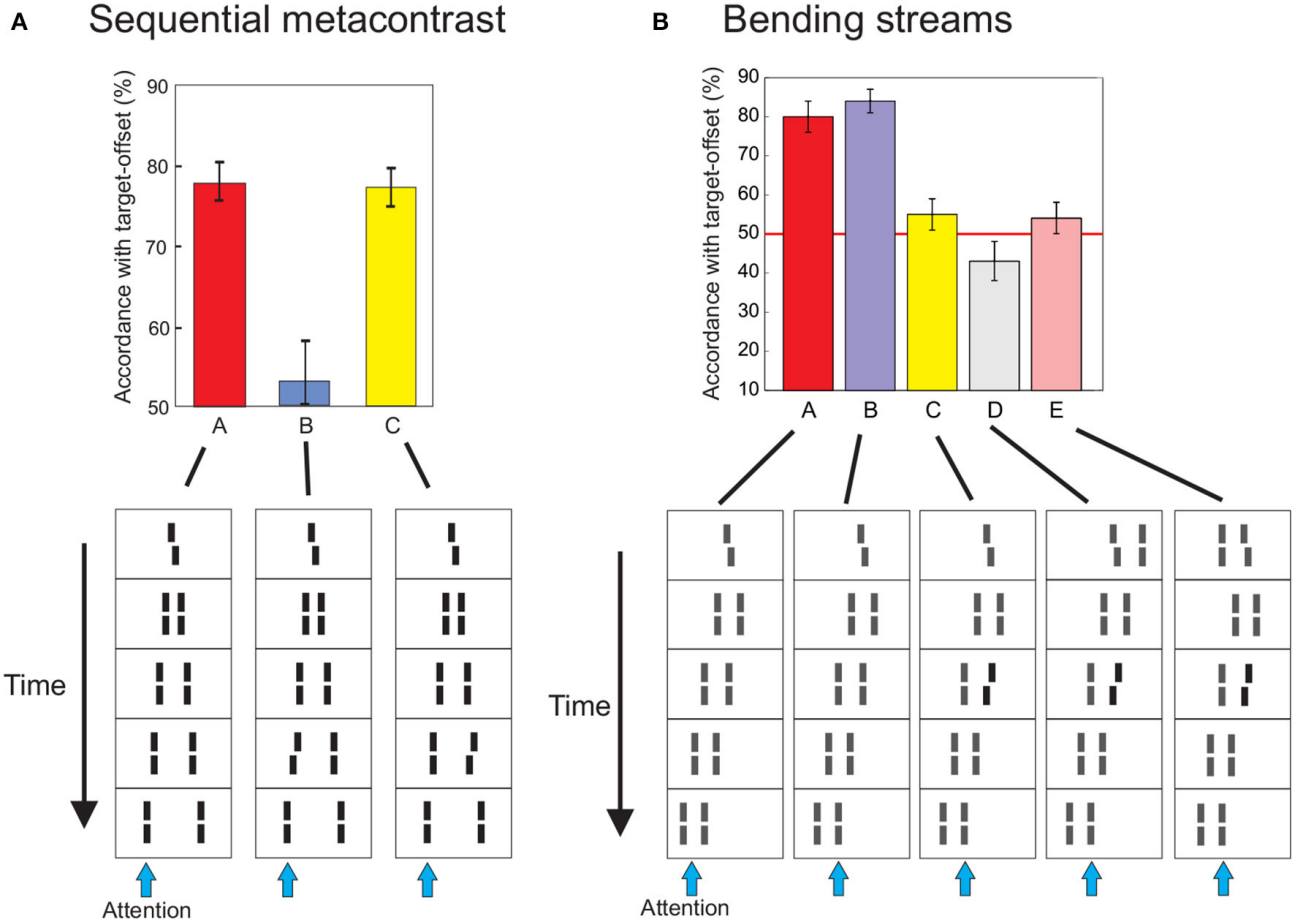
**(A)** Feature integration also occurs when elements are presented at different retinotopic locations. A central vernier, offset either to the left or right, was flanked by *aligned* verniers after short ISIs leading to the percept of an expanding motion stream. Even though the vernier is invisible by metacontrast masking, its offset is perceived at the aligned verniers. To give quantitative expression, we asked observers to attend to the left (or right) motion stream and report the perceived offset. The task is natural to the observers since the vernier offset is inherited to the flanking lines and thus clearly visible. Adding an opposite offset (anti-offset) to one flanking line, leads to integration as in feature fusion. Integration of vernier and flanker offsets occurs only in the attended stream (blue arrows indicate the attended stream). When the offset of the flanker is presented in the non-attended stream, it is not integrated with the vernier offset (C). **(B)** Integration depends on flexible grouping. We presented “bending streams” leading to similar integration of the vernier offset when observers attend to either the right or left stream because the vernier is inherited to both streams (A,B). In (C) we added an anti-offset to the second flanker of the right stream, i.e., at the same spatial location as the central vernier. The vernier and flanker offset integrated. Next, we added single lines to the vernier, which changed the spatio-temporal grouping and, accordingly, feature integration (D, E). If the line is presented to the left of the vernier, the vernier is inherited to the left stream only and hence its offset is integrated in the left stream. For this reason, the vernier offset is not integrated in the right stream and thus the flanker anti-offset dominates performance when observers attend to the right stream (performance is below 50% because the flanker offset is always offset in the opposite direction than the vernier). (E) When the single line in the first frame is placed to the left of the central vernier, grouping and hence performance changes. The vernier offset is now exclusively integrated in the right stream and thus vernier and flanker offset cancel each other. Figures adopted from Otto et al. (2006).

We presented a left or right offset vernier as the first element, followed by a sequence of *aligned* verniers (Figure 3). Two expanding streams of lines are perceived originating from the center of the screen (sequential metacontrast, Otto et al., 2006). The first vernier is rendered unconscious. To show this, we presented the sequence with and without the vernier in two subsequent intervals. Observers indicated whether the vernier was present or absent (direct measure). Performance was close to chance level. Surprisingly, however, the vernier offset is visible at the flankers even though the flankers are aligned and the vernier itself is invisible (feature inheritance; Herzog and Koch, 2001; Sharikadze et al., 2005). In quantitative experiments, observers could well discriminate whether the offset is to the left or right (second direct measure; Figure 3). When one or more of the flanking lines are offset themselves, the target and flanker offsets integrate as in feature fusion with two verniers presented one after the other at the same location. When the offsets are in the same direction, performance improves. When the offsets are in opposite direction, they cancel each other out. Sequential metacontrast therefore shows that features are integrated across different retinotopic locations. Integration is almost linear (Otto et al., 2006, 2008). We propose that the first vernier and all flankers are interpreted as being part of a whole (the motion of one line) and, for this reason, the individual elements are rendered invisible. Similar feature inheritance like effects, called feature transposition or feature attribution^2^, have been reported also in other paradigms (Werner, 1935; Stewart and Purcell, 1970; Wilson and Johnson, 1985; Hofer et al., 1989; Herzog and Koch, 2001; Parkes et al., 2001; Enns, 2002; Nishida et al., 2005; Öğmen et al., 2006; Otto et al., 2006, 2008).

In the sequential metacontrast paradigm the target itself is completely rendered unconscious (direct measure), i.e., observers cannot even tell whether or not there is a vernier. In most other masking paradigms this is not the case, as mentioned above. In these paradigms, even though the target is subjectively invisible, there are still some motion and luminance cues allowing for target detection. As in feature fusion, we have shown that the unconscious vernier changes the perception of a second direct measure, namely the inherited vernier offsets, visible at the flanks. Hence, our paradigm shows unconscious processing that is independent of pre-activations of the motor-cortex, as in masked priming (e.g., Eimer and Schlaghecken, 2003). The invisible vernier target offset is even integrated with offsets of flanking elements, which can be presented more than 400 ms after target onset, which is much longer than the reaction times in mask priming experiments. We have recently shown that feature fusion even precedes motor-priming (Grainger et al., 2013). As in the TMS experiment above, the sequential metacontrast paradigm shows that features of invisible elements can persist in the human brain for substantially long times.

As in the case of vernier fusion at one retinotopic location, non-retinotopic integration follows “flexible” grouping rules as demonstrated in Figure 3B. As before, we propose that the human brain first carefully analyzes complex motion trajectories and, then, integrates features along motion streams. Changes in grouping lead to changes in integration.

Such a mechanism makes sense from an ecological perspective. At night, a car is running through a modestly illuminated street. The luminance (color of car ⋆ light shining on car) changes strongly from instance to instance because of the distance between street lights, shadows, reflections, etc. In addition, each photoreceptor receives information only for a very short time. Hence, averaging across the motion trajectory is a first step to compute the “true” color of the car, independent of the illumination. In addition, the car may disappear when passing through a shadow. During this period, averaging needs to stop and only continue when the car reappears. Similarly, we have shown that the brain stores vernier offset information in a short term memory when the verniers are occluded. After re-appearance the memory re-opens and continues averaging vernier offsets (Scharnowski et al., 2007b).

## 3. Discussion

### 3.1. Invisibility under different masking types

How a visible stimulus becomes invisible due to the presence of another stimulus is the focus of visual masking research (Bachmann, 1994; Breitmeyer and Öğmen, 2006). Visual masking is not a unitary phenomenon and several different types of masking have been identified. First, one can classify masking into two broad types, masking by light and masking by pattern (Breitmeyer and Öğmen, 2006). In masking by light, the mask consists of a uniform light field. The loss of target visibility in this masking type can be explained by the aforementioned low-level irreversible factors related to dynamic range adjustments. Masking by pattern can be divided into two broad types, pattern masking by noise and pattern masking by structure (Breitmeyer and Öğmen, 2006). In pattern masking by noise, the mask is a noise pattern, such as an array of randomly placed dots, which do not bear structural similarity to the target. Here, with appropriate timing, the target and mask integrate into one conglomerate thereby hampering the visibility of the target (Breitmeyer and Öğmen, 2006). However, we claim that a different process takes place when both the target and mask have structure and the visual system forms Gestalts both in space and time (Ternus, 1938). We suggest that this process is flexible in that it can lead to integration or segregation in time depending on the context. Thus, invisibility arising from temporal integration is not a limitation of visual system due to its slow dynamics, but rather a purposeful computation as part of selecting the best Gestalt across space and time.

More recently, it has been proposed that four-dot masking, also known as object substitution masking or common onset masking, is a fundamentally different type of masking (Enns and Di Lollo, 1997, 2000; Di Lollo et al., 2000). Even though there is a debate whether this is a truly different type of masking (Breitmeyer and Öğmen, 2000; Di Lollo et al., 2002; Francis and Hermens, 2002; Breitmeyer and Öğmen, 2006) it is important to highlight some similarities and differences between two models that are based on this paradigm and our approach. These accounts, known as object substitution or updating masking, are based on object-level representations (Enns and Di Lollo, 1997, 2000; Di Lollo et al., 2000; Lleras and Moore, 2003; Moore and Lleras, 2005; Pilling and Gellatly, 2010). They propose that the invisibility of a target results from the substitution or updating of the target representation by the mask representation during the iterative activities set up in cortical reentrant (feedback) loops. Under the four-dot or common onset masking conditions, the features of the invisible target are incorporated into the mask. Thus, according to these theories, feature attribution is clearly linked to the process of masking by the common process of object substitution, which occurs during reentrant object updating^3^. Hence, these approaches predict a strong correlation between feature attribution and masking. On the other hand, in our approach we predict a dissociation between visual masking and feature attribution (Öğmen, 2007; Öğmen and Herzog, 2010; Öğmen et al., 2006; Otto et al., 2006; Noory et al., under review). We tested these contrasting predictions in a study where we determined the correlations between feature attribution, masking, and motion (Breitmeyer et al., 2008). We found that feature attribution correlated strongly with motion, but not with backward masking (Breitmeyer et al., 2008). Thus, these results argue against object substitution or updating models and support a model wherein feature attribution and masking serve complementary but distinct roles (Öğmen, 2007; Öğmen and Herzog, 2010).

### 3.2. The many mechanisms of invisibility

**Figure 4:**
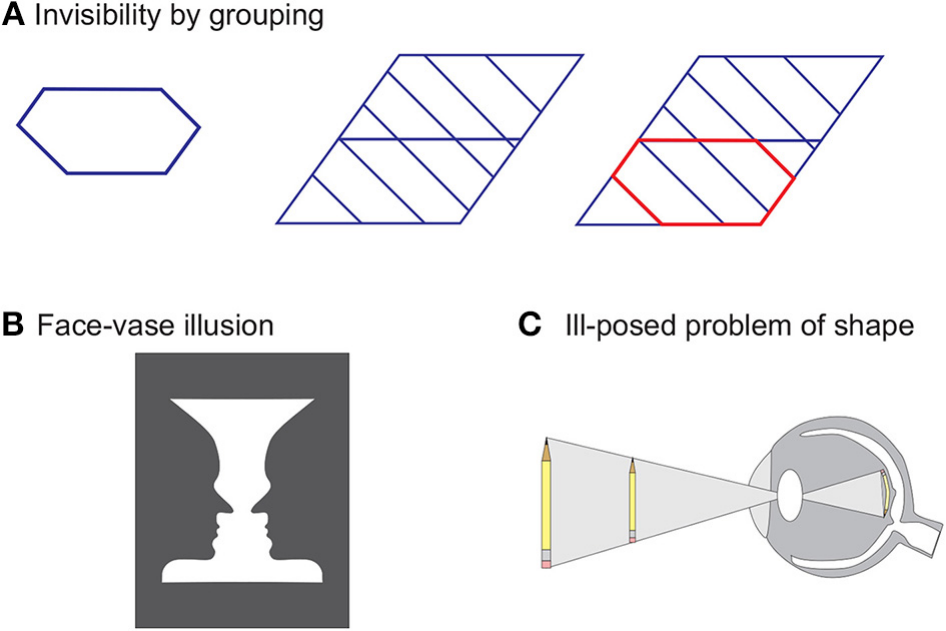
**(A)** The hexagon on the left is clearly visible. The very same hexagon is hard to find in the shape in the middle because adding further elements leads to a very different perceptual organization. A simpler overall shape is perceived and the hexagon, as a sub-shape, is rendered invisible (solution on the right, shape in red). Adopted from Aydin et al. (2011). **(B)** Either a face or a vase is perceived. It is impossible to see both interpretations at the same time (Rubin, 1915). **(C)** The retina codes the external world by a 2-D representation. Hence, the brain needs to infer the third dimension. For example, there are infinitely many pens (only two are shown) that give rise to the same retinal image. To infer which pen is presented, the human brain needs to estimate two unknowns, namely the size and distance of the pen. This is an ill-posed problem, i.e., there is no unique mathematical solution, since only one value is available, namely the retinal size of the projection of the pen.

Invisibility, as a goal and interpretation, is ubiquitous, occurs on many levels wherever the human brain needs to solve the ill-posed problems of vision (see below). For example, the hexagon on the left of Figure 4A is clearly visible. The shape in the middle contains this hexagon as well, but due to the grouping of the oblique lines into a global but simpler pattern, the hexagon becomes invisible. Highlighting the hexagon by a color difference leads to another perceptual organization where the hexagon becomes visible again. Other well known examples are binocular rivalry and ambiguous figures (Figure 4). There are many other “flexible” mechanisms that can render a target invisible. For example in change blindness, a target, even though presented for extended amounts of time, is invisible because of a lack of attention (for reviews, see Simons and Rensink, 2005; Simons and Levin, 1997). Moreover, it has been shown that visibility of the target in masking is influenced by the task. In particular, differences are found when participants are asked to report the luminance of the target, compared to when they are asked to respond as quickly as possible to the location of the target (Fehrer and Raab, 1962; Schiller and Smith, 1966; Öğmen et al., 2003). Hence, visibility of the brightness of a stimulus may be impaired in different ways than the visibility of the spatial position of a stimulus, because the two aspects of the stimuli may be represented differently (Breitmeyer and Öğmen, 2006).

However, we do not claim that invisibility is always interpretation. To the contrary, as mentioned above, low level limitations can render a target irreversibly invisible, for example, when the mask is of high luminance.

So far, we have considered grouping from a Gestalt psychology point of view. At the moment, we do not want to speculate on the neural mechanism that underlie interpretation and grouping but just like to mention that more and more neurophysiological evidence (Roelfsema and Houtkamp, 2011; Wannig et al., 2011) is becoming available.

### 3.3. Why does invisibility occur at all? why is visibility unique and not bayesian?

The idea of invisibility as a goal and interpretation can be traced back to the Gestaltists who forcefully demonstrated that our perceptual experience is *not* a simple collection of stimulus elements but instead is based on the formation of wholes (Gestalts). The visual input consists of a staggering amount of elementary stimulations (the pixels of an image or photo-receptor activations). Because there are “infinitely” many possible ways these stimulations can combine together, “laws” such as proximity, similarity, and good continuation were introduced to limit the number of possible solutions. Guided by classical physics, according to which the state of the world is both unique and determined by a minimum energy principle, the Gestaltists formulated the law of Prägnanz, which states that only the “simplest” solution becomes visible and all other solutions remain invisible (Koffka, 1935).

When different solutions are of *similar* simplicity as in binocular rivalry or ambiguous figures, such as Rubin's face/vase example, a small difference, such as the locus of attention, can cause switches between the prevailing solutions. Nevertheless, still only one solution is visible at a moment (Figure 4B).

Why is there only one interpretation at one moment of time? There are infinitely many pens that give rise to the very same retinal image in Figure 4C. Hence, there are infinitely many interpretations. This is the classic ill-posed problem of size perception, which is just one example of the many ill-posed problems of vision. Hence, for a unique percept, infinitely many interpretations of one retinal image need to be invisible. For this reason, in ambiguous figures and binocular rivalry, there are not two, but infinitely, many rivaling interpretations, of which only two or a few *can* become visible. Hence, rendering information invisible is a normal feat of the human brain. We like to argue that (in)visibility in masking paradigms often occurs for the same (normal) reasons.

From a philosophical and computational point of view, one may ask why is it that the brain has to select only one of the many possible interpretations for our phenomenal experience. An alternative view would be a Bayesian type of approach where all alternative interpretations become visible with some indication of their likelihood. As mentioned, the Gestalt psychologists were inspired by classical physics and incorporated into their law of Prägnanz the notion that the state of the world is *unique* and is determined by a minimum energy state (Koffka, 1935). We would like to add the following arguments: First, even for stimuli as in the pen example of Figure 4C, there are infinite or close to infinite possible interpretations and therefore making all of them visible is not feasible. One may argue that, perhaps one can pick the top *n* most likely interpretations and make this smaller subset visible. Even if we were to pick a small subset at a given time, the combinations across time would still grow toward infinity. Let's assume that a selection is made also in time so as to always limit the number of visible percepts to *n*. The problem with this is that it will break down the unity of consciousness in that the subject will be living in “parallel realities” where different combinations in different percepts can lead to different learning and experiences, whose number and diversity can grow rapidly over time. Thus, we suggest that a unique visible configuration is chosen to maintain the unity of consciousness.

### 3.4. The role of volitional control

We do not claim that interpretation is under the control of volition. To the contrary, integration within the motion streams of the sequential metacontrast paradigm is mandatory (Herzog, 2007). Observers can only choose to which stream they want to attend to but they cannot attend to one line individually (see also Otto et al., 2006, 2008). In addition, integrated features are usually metamers, i.e., it is impossible to judge whether a perceived vernier offset comes from one or several verniers (Herzog and Koch, 2001; Scharnowski et al., 2009; Hermens et al., 2010). The same mechanisms of attention can explain why masking can be influenced by the task (for a discussion, see Breitmeyer and Öğmen, 2006) and individual differences (e.g., Albrecht et al., 2010).

Neither do we claim that invisibility by interpretation does not occur stereotypically. The very same stimulus, presented over and over again, can lead to the very same percept depending on the rules of grouping of visual scenes. In addition, a stimulus may lead to the very same percept for different observers and is thus less variable than, for example, the interpretations of poems.

### 3.5. Methodological and conceptual implications for (un)consciousness research

Our results have strong methodological implications for (un)consciousness research. For example, observers cannot report the offset direction of the central vernier in the sequential metacontrast paradigm (Figure 3), simply because the vernier is invisible. Still, the offset is, unexpectedly from a retinotopic point of view, visible at the non-offset flanking lines. Hence, visibility may go unnoticed depending on the task of the observers. In fact, in the early reports such feature inheritance like effects were considered as nuisances and potential sources of artifacts of metacontrast masking (see Hofer et al., 1989; Stewart and Purcell, 1970). This is important for unconsciousness research, where the difference in the indirect measure between conscious and unconscious conditions is often rather small in the range of a few milliseconds of reaction times (Naccache et al., 2002). It hence may be possible that observers base their indirect measure decisions on inherited features, but not for the direct measure. In this sense our paradigms offers an interesting alternative to the classic direct-indirect measures by determining unconsciousness with two direct measures, which in addition both measure accuracy in a similar way and have similar sensitivity.

Our results challenge many models of consciousness. For example, invisibility is often proposed to occur because of a lack of recurrent processing (Lamme and Roelfsema, 2000; Dehaene et al., 2001; Naccache et al., 2002). However, in the sequential metacontrast paradigm flankers integrate with the vernier even when presented 300 ms after vernier onset implicating recurrent processing because of the long difference in presentation times and the integration process. In addition, the vernier offset is visible at the flankers and hence needs to be processed by recurrent processing. However, why is then the vernier itself not visible? Why is the vernier spared from recurrent processing but not its offset? These kinds of observations are only possible, because we used direct measures, which allow us to test for long term effects of invisible features. This is impossible with an indirect measure such as speeded reactions times.

## 4. Summary

We have shown that the visibility of an element can crucially depend on how it is grouped with other elements, even in situations, which were previously attributed to lower limitations. In these cases, we propose that the human brain carefully registers the features of all incoming elements. As our TMS experiments have shown, these features can be stored for a substantial time of more than 400 ms. During this period, the brain collects information to compute which elements belong together and then integrates features into a coherent percept. Visibility and invisibility are just outcomes of this process. Hence, we have argued that much of the invisibility found in perception can be the result of purposeful selections made by the brain. However, there are many other aspects, including low level limitations and attentional selection, which are as crucial for visibility. Hence, it is important to clarify in each paradigm and situation to which extent each of these factors are in operation.

### Conflict of interest statement

The authors declare that the research was conducted in the absence of any commercial or financial relationships that could be construed as a potential conflict of interest.

## Footnotes

^1^Note that if the offsets of the verniers are large, one can see four line segments instead of two, while for intermediate offsets, motion is perceived, see Figure 1 in Scharnowski et al. (2007b). Here the vernier offsets are kept small enough so as to have fusion of the two verniers.

^2^These terms emphasize different aspects of the phenomenon. Feature inheritance emphasizes the changes occurring to the mask, feature transposition emphasizes the transfer of the feature from the target to the mask, while feature attribution emphasizes the interpretive process where the target and mask are linked to each other via motion grouping and thus share their features.

^3^It is important to note that under four dot masking paradigm, no masking occurs when attention is not divided and directed to the target. From this point of view, object substitution is not applicable to the results shown here because in the presently discussed work the target location is predictable and receives full attentional resources. The object updating account may be applicable depending on specific mechanisms of object updating, which, unfortunately, remain unspecified.
